# Can a novel constructivist theory-informed feedback intervention reduce prescribing errors ? A pre-post study

**DOI:** 10.1186/s12909-023-04095-6

**Published:** 2023-03-07

**Authors:** Ian Coombes, Peter Donovan, Brooke Bullock, Charles Mitchell, Christy Noble

**Affiliations:** 1 School of Pharmacy, University of Queensland, 20 Cornwall St, Woolloongabba, QLD 4102 Australia; 2 Clinical Pharmacology. Royal Brisbane and Women’s Hospital, Butterfield St, Herston, QLD 4029 Australia; 3 School of Medicine, University of Queensland, Herston, QLD 4029 Australia; 4 Advanced Pharmacist. Gold Coast Hospital and Health Service, 1 Hospital Boulevard, Southport, QLD 4215 Australia; 6 Clinical Learning and Assessment Lead, Academy for Medical Education, Medical School, The University of Queensland, Herston, QLD 4006 Australia; 7 Pharmacy Department, Royal Brisbane and Women’s Hospital, Butterfield Street, Brisbane, QLD 4029 Australia

## Abstract

**Context:**

Medical interns (interns) find prescribing challenging and many report lacking readiness when commencing work. Errors in prescribing puts patients’ safety at risk. Yet error rates remain high, despite education, supervision and pharmacists’ contributions. Feedback on prescribing may improve performance. Yet, work-based prescribing feedback practices focus on rectifying errors. We aimed to explore if prescribing can be improved using a theory-informed feedback intervention.

**Methods:**

In this pre-post study, we designed and implemented a constructivist-theory informed prescribing feedback intervention, informed by Feedback-Mark 2 Theory. Interns commencing internal medicine terms in two Australian teaching hospitals were invited to engage in the feedback intervention. Their prescribing was evaluated by comparing errors per medication order of at least 30 orders per intern. Pre/baseline (weeks 1–3) were compared with post intervention (weeks 8–9). Interns’ baseline prescribing audit findings were analysed and discussed at individualised feedback sessions. These sessions were with a clinical pharmacologist (Site 1) and a pharmacist educator (Site 2).

**Results:**

Eighty eight intern’s prescribing over five 10-week terms was analysed from two hospitals. The frequency of prescribing errors significantly reduced at both sites after the intervention, across all five terms (p < 0.001).There were initially 1598 errors in 2750 orders (median [IQR] 0.48 [0.35–0.67] errors per order) and after the intervention 1113 errors in 2694 orders (median [IQR] 0.30 [0.17–0.50] errors per order).

**Conclusion:**

Our findings suggest interns’ prescribing practices may improve as a result of constructivist -theory learner centred, informed feedback with an agreed plan. This novel intervention, contributed, to a reduction in interns’ prescribing errors. This study suggests new strategies for improving prescribing safety should include the design and implementation of theory-informed feedback interventions.

**Supplementary information:**

The online version contains supplementary material available at 10.1186/s12909-023-04095-6.

## Introduction

Medical interns (interns) find prescribing challenging and many report lacking readiness when commencing clinical work [1–4]. This lack of readiness results in prescribing errors and increases risks to patient safety. Whilst prescribing errors are complex and involve multiple stakeholders, [5–7] factors contributing to prescribing errors include the prescribers’ knowledge and skills, supervision, patient factors, and prescribing system failures. [2, 7, 8] Common strategies to improve prescribing safety include educating medical students and junior doctors, [9, 10] electronic prescribing systems with and without clinical decision support [11, 12] and clinical pharmacy services [13].

Another approach to improve prescribing performance is through feedback [12]. Interns value feedback from consultants and pharmacists as it promotes learning about prescribing. Prescribing feedback, in clinical settings, does not occur in a regular and systematic way [14, 15]. Instead, hospitals tend to undertake de-identified large scale audits as part of quality assurance programs and for purposes of accreditation without evidence of sustained impact on clinician performance unless feedback is targeted at individual prescribers [16]. In contrast, pharmacists’ feedback largely focuses on identifying, communicating and rectifying individual errors with little guidance on how to improve interns prescribing performance [1].

There is growing attention to enhancing prescribing safety through feedback interventions. However, the approaches to feedback interventions have been diverse in their design (e.g. provision of feedback information with or without discussion) and their impact on improving practice have been inconsistent. For example, a recent intervention in the United Kingdom (UK) aimed to reduce prescribing errors by improving feedback to junior doctors [17]. Despite implementing strategies to augment pharmacist prescribing feedback using emails with details of their errors, no changes in the rate and severity of prescribing errors were observed [13]. The feedback intervention described used a one-way feedback process, where pharmacists told the junior doctors about their medication error. In another feedback intervention study [18], the feedback intervention was described as a pharmacist led discussion about the frequency, type and severity of prescribing errors that had occurred in a three week period, and supplemented with education, resulted in a reduction in prescribing errors. Another feedback intervention study focussed on improving antimicrobial prescribing, which conducted feedback workshops whereby junior doctors were provided with personalised feedback information, followed by group discussions about prescribing and its challenges [19]. This feedback intervention improved antimicrobial prescribing. However, none of the three studies described the feedback theory informing the intervention design. This is important because feedback can be theoretically understood in different ways [20], and it is well recognised that one-way feedback processes are less effective, in terms of improving performance [21].

In contrast, a pilot feedback intervention study where pharmacists provided individualised feedback (based on principles, not theories, described in medical education literature [22, 23]) to junior and senior doctors in an acute UK hospital generated a significant reduction in prescribing errors [24]. Finally, a recent study, informed by self-regulated learning and using pharmacist-led, video-stimulated feedback contributed to a significant reduction in prescribing errors by 16 doctors [25]. Overall, the impact of several feedback studies on prescribing have been inconsistent.

A recent review of ‘audit and feedback’ interventions sheds some light on why these variations in effects occur, that is, there is tendency to design feedback interventions based on “intuitive, non-theoretical ideas about what might work”(p.2) [26]. The authors argued that to maximise the effects of feedback interventions and improve consistencies, a theory-informed approach to feedback intervention design is required [26].

## Aim

The aim of this study was to determine the effects of a constructivist theory informed feedback intervention on intern prescribing behaviour. To achieve this aim, we designed, implemented, and evaluated a theory-informed self and peer feedback intervention, in two sites, and measured prescribing errors before and after the intervention.

## Methods

### Overview of study design

We designed, implemented and evaluated a constructivist theory informed feedback intervention, with the overall goal to observe the effect on prescribing practice [27]. Drawing on contemporary education and health profession’s education literature, we identified Feedback Mark 2 Theory, based on constructivist learning theory [28], to inform our intervention design. From this perspective, feedback is described as “a process whereby learners obtain information about their work in order to appreciate the similarities and differences between the appropriate standards for any given work, and the qualities of the work itself, in order to generate improved work”(p. 7, [21]). Aligned to this theory, the feedback intervention was designed and implemented in three phases (see Table 1) to five consecutive cohorts of medical interns across successive 10 to 12 week terms, at two teaching hospitals. The intervention included self-assessment, pharmacist review, medication chart audit and individualised feedback (with either a clinical pharmacologist or clinical pharmacology registrar at one site or a pharmacist with medical education expertise at the other site).

### Settings and participants

The study was conducted in southeast Queensland, Australia at a 929-bed teaching hospital (Hospital 1) and a 750-bed tertiary teaching hospital (Hospital 2). All interns in internal medicine terms were invited to participate. Both hospitals have established intern training programs, with a total 180 interns who rotate through various disciplines including internal medicine, surgery, emergency and one or two specialty areas. At both sites, like other hospitals in Australia, most inpatient prescribing occurs on the National Inpatient Medication Chart (NIMC). The interns had access to all online prescribing decision support tools through hospital networks and each medical team had the services of designated clinical pharmacists.

**Table 1:** Overview and timeline of study design for each Term

Phase	Timing in term	Feedback Mark 2 principles	Intervention component	Data collection
1	Start of each term	Orientation to standards of prescribing and purpose of feedback	Participants briefed on intervention and provided “Resident Prescribing Competency Evaluation and Feedback, Safety and Quality Development Tool” (Appendix 1)	n/a
	Weeks 1 to 3	Activity 1	Interns engage in day-to-day prescribing	Prescribing outcomes (Appendix 2)
2	Weeks 4 to 5	Learner judges work	Individualised feedback session (including intern self-assessment) and generation of development plan for improving the prescribing of the learner informed by ward pharmacist assessment and prescribing outcomes audit with: • Site 1 – Clinical Pharmacologist/ Clinical Pharmacology Registrar • Site 2 – Senior clinical pharmacist (medical educator)	Intern self-assessment of prescribing practice (Not reported on in this manuscript.)
		Learner asks for specific feedback		
		Others judge work		
		Compare judgement		
		Plan for improved work		
3	Weeks 8 to 10	Activity 2	Interns engage in day-to-day prescribing	Prescribing outcomes (Appendix 2) Intern evaluation of engaging in intervention (questionnaire)
		Evaluation of effects	Repeat of prescribing audit	

**In Phase 1** at the start of each of 5 sequential internal medicine terms, the interns were orientated to the purposes of feedback; that is, to generate safe and effective prescribing practices, and to the ‘standards of work’ using “Resident Prescribing Competency self and Peer Evaluation and Feedback, Safety and Quality Development Tool” which incorporates the key standards for safe prescribing (See Appendix 1). The prescribing competencies, based on National Safety and Quality Medication Safety Standards [29], included improving patient identification documentation, and appropriate prescription of venous thromboembolism prophylaxis, antimicrobial stewardship, medication history taking and reconciliation and ceasing medication appropriately. Also, interns were given the “Recommendations for Terminology, Abbreviations and Symbols used in the prescribing and Administration of Medicines” [30].

The interns then prescribed (Activity 1 in Table 1) for three weeks after which, to promote the initial component of self-regulation [21], they were invited to self-assess their prescribing practices by completing the Resident Prescribing Competency Evaluation and Feedback, Safety and Quality Development Tool. This tool uses a four-point Likert scale to assess 14 key prescribing competencies (see Appendix 1). During the same initial three weeks, the intern’s prescribing was observed and assessed by the ward-based pharmacist using the tool based on National inpatient medication Chart (Appendix 2). The details of the interns’ self-assessment and ward-based pharmacist assessments will not be presented here.

**In Phase 2**, during weeks 4 to 5 of the term, a feedback session was conducted with each intern informed by the (1) initial audit (see below), (2) their self-assessment and (3) the ward pharmacist assessment at a time convenient for both the intern and feedback facilitator.

During the feedback sessions, typically lasting 30 to 45 min, the facilitator and intern compared the audit findings presented as a summary of individuals prescribing performance along with specific examples using photocopies of medication charts. The interns’ self-assessment and the ward pharmacists’ assessment were also discussed and together a plan for improved work was generated. It was anticipated that this approach gives specific, individualised data on the ability to complete the various tasks of prescribing and engages the intern in a process of self-evaluation likely to enhance their ability to assess and then make decisions about the quality of their prescribing [31].

In **Phase 3** - the evaluation of effects: the interns’ subsequent prescribing practice (Activity 2) was re-audited to determine the impact of the feedback on the number of errors per order and details of the prescribing error audit (see Appendix 2). The interns and ward pharmacists were asked to repeat their self-assessment during the last two weeks of the term.

### Interns’ perception of intervention

The interns’ experiences of the intervention were explored using a validated paper-based satisfaction survey [32] which includes Likert scale and open questions [33]. Responses were scored on a 7- point Likert scale (1 = strongly agree, 7 = strongly disagree). Responses from the open questions were analysed thematically [34, 35] and Likert items were analysed using descriptive statistics.

### Data collection

Data were collected on the number of patients, orders, prescribing errors and previous Adverse Drug Reaction (ADR) documentation. Prescribing errors were identified by our research assistant (pharmacist) who conducted daily review of medications charts, identifying prescriptions from the participating intern prescriber. Medications errors were defined as, any order that could result in the administration of wrong drug, form, route, dose, frequency, to the wrong patient, according to the standard NIMC audit conducted across multiple sites in Australia. Each participant’s prescriptions were selected by convenience sampling, but a minimum of 30 orders was mandated before and after the intervention, to improve the representativeness of the sample.

### Data analysis

Data analysis was conducted using Stata (version 12.1, StataCorp Texas, United States). The number of errors per medication order, per individual participant were calculated before and after the intervention. Medians and interquartile ranges are presented as these data were not normally distributed. Differences in errors per order before and after feedback were assessed with the Wilcoxon signed rank test for paired data. Subgroup analysis compared error rates at each of the two sites involved in the study and for each of the five medical terms to assess whether gained experience throughout the intern year had any influence on the impact of the intervention. Wilcoxon signed rank sum test was used to compare differences in the improvement in error rates at the two different sites. For each domain of prescribing outlined on the “Resident Prescribing Competency Evaluation and Feedback, Safety and Quality Development Tool,” the overall proportion of errors (of the total opportunities for error – i.e. orders) was determined and compared using Chi-square.

In the analysis of the pharmacist and medical officer assessments of prescribing competency, the results were presented as the proportion of participants who answered Agree, Neutral or Disagree for each question.

## Results

### Participants

89 intern prescribers (51 from Hospital 1 and 38 from Hospital 2) were recruited during their internal medicine terms between March 2016 and March 2017 and 88 completed the study.

### Prescribing outcomes

All 89 prescribers engaged in all three phases of the intervention. 88 had both pre- and post-intervention data available for analysis.

In total, 2750 orders were reviewed pre-intervention (median 30; IQR [30–35] per prescriber, 2168 regular orders, 582 as required[prn]) and 2694 post-intervention (median 31 [31–33] orders per prescriber, 2163 regular, 531 prn).

There were 1598 errors pre-intervention and 1113 post-intervention. Overall, there was a 38% reduction in prescribers’ median errors per order from 0.48 [IQR 0.35–0.67]to 0.30 [IQR 0.17–0.50] (p < 0.0001) – see Table 2. Despite different professions of ‘feedback providers’ (i.e. a pharmacist, a clinical pharmacologist or a registrar) at each site, there were no differences in the improvement in prescribing performance seen between the two sites (p = 0.93).

**Table 2:** Demographics Median Errors per order per prescriber from observational audit of NIMC

	Pre	Post	Improvement	P value
Number prescribers	89	88	-	-
Number orders	2750	2694	-	-
Number errors	1598	1113	-	-
Errors per order - median [IQR]	0.48 [0.35–0.67]	0.30 [0.17–0.50]	0.17 [0.02–0.33]	< 0.0001
Site 1 only errors per order - median [IQR]	0.44 [0.3–0.61]	0.22 [0.10–0.37]	0.17 [-0.03-0.35]	0.0001
Site 2 only errors per order - median [IQR]	0.48 [0.35–0.67]	0.3 [0.17–0.50]	0.15 [0.05–0.33]	0.0006

**Fig. 1:**
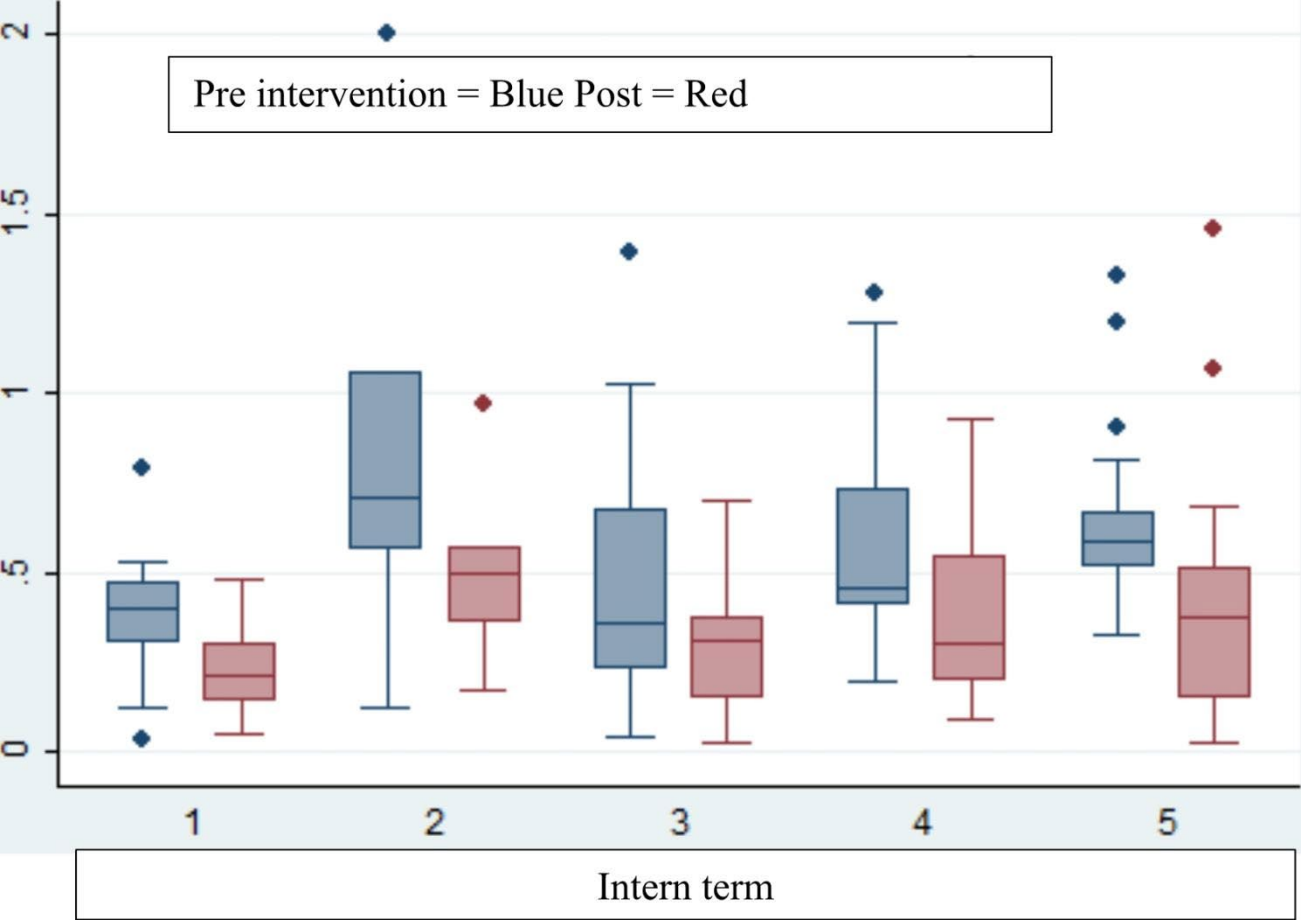
Box and Whisker plot Median errors per order per prescribe. In Terms 1, 3, 4, 5 over the intern year the reductions in error rates from before the intervention (blue boxes) to after (red boxes) were significant (p < 0.05) n = 88 interns

Figure 1 shows the errors per order over the five terms and before and after the intervention. Prescribing errors were reduced after the intervention in each cohort. Whilst not designed or powered to show differences between the cohorts, there were similar reductions in error rate per order across the five terms.

Over the five terms, there were similar error rates prior to the intervention.

### Intern evaluation of the prescribing intervention

Sixty-five interns (73%) completed the evaluation survey. Overall, of those that provided feedback the acceptability of the intervention was very high, where most of the interns either strongly agreed or agreed that the intervention offered was fair, positive, not too onerous, helpful and useful.

Forty-two (47%) interns also provided qualitative comments. Almost 70% (n = 29) described the experience as positive with 50% (n = 21) explicitly noting the benefits of having individualised feedback based on practice examples; 33% (n = 14) stated that they valued guidance on how to improve their prescribing practice. A few participants (n = 4) felt that more examples of prescribing practice were required to make a comprehensive judgement on their prescribing performance. Only one of 42 interns described the intervention as challenging and difficult to incorporate into their day; more than 10% of interns noted the intervention was time consuming.

## Discussion

We found a median of 38% reduction in prescribing errors per order. This finding suggests that our theory informed feedback intervention influenced prescribing behaviour during their internal medicine term. The improvement was observed in the five cohorts of interns over the year in two separate sites. This finding suggests that usual medical supervision, interaction with pharmacy staff and generic prescribing audits undertaken as routine safety and quality activities, in preceding terms (from Term 2 onwards), did not impact on prescribing. These findings contribute to an emerging body of evidence that theory-informed feedback interventions may improve prescribing behaviour [25].

We believe that three aspects of our intervention may have contributed to its impact. Firstly, the intervention was specifically designed to create an opportunity for interns to make sense of their errors through discussion and to construct strategies to improve their prescribing. This constructivist approach is advantageous as prescribing feedback practices in hospital settings such as clinical pharmacy discussions on wards tend to be error focussed and ad hoc [1, 14]. Our findings resonate with other studies [25, 33] emphasising the value of feedback on clinicians’ performance during protected time, away from busy clinical areas. Also, the focus on improving prescribing, perhaps explains why the intervention had a high degree of acceptability with the interns.

Secondly, our findings extend the work of other studies [17], with similar participants (i.e. junior doctors) and settings (hospital), where feedback was provided as fortnightly “good prescribing tip” emails to all intern prescribers. Whilst other intervention studies that have demonstrated improved prescribing outcomes, used simulation [36, 37] or video methods [25]. The advantage of our intervention is that it was grounded in practice, where prescribing occurs, and so accounts for the highly complex and unpredictable context of prescribing in hospitals [38, 39].

Thirdly, a surprising observation was that there were no difference in prescribing safety outcomes when feedback was provided by a pharmacist or a clinical pharmacologist, although the study was not powered to assess for any difference related to who provided the feedback. This may imply that it is the theory-informed feedback process, rather than the role or profession of the individual providing the feedback that has an impact on junior doctors prescribing practice. Having pharmacists involved with the feedback helps develop interprofessional respect and trust [14]. This structured intervention provides an opportunity for systematic prospective and proactive formative feedback, a shift in the usual feedback from pharmacists in practice, which is often random, “on the run” and ad hoc often based on the pharmacist detecting a prescribing error.

### Limitations of the study

Whilst there was no control group in our study, the interventions were conducted across two sites over five separate terms with five separate cohorts at each site which will reduce validity threats experienced by single-group pre-test-post-test studies [40]. Another limitation is that we used documentation (i.e. completion of NIMC) as a marker of prescribing performance. However, prescription charts are only one form of monitoring prescribing performance and we cannot assume, as argued by others, that this indirect outcome (i.e. fewer prescribing errors) translates to a reduction in medicine related harm and improved quality of care for patients [41]. However, our study was not powered to assess these patient-centred outcomes. Whilst we have used outcomes which are generally agreed upon to be important determinants of safe and effective prescribing, designing feedback studies that relate feedback to measurable patient outcomes is inherently difficult [41].

Another potential limitation was that we only evaluated the effect on prescribing tasks (i.e. prescribing errors), the learners’ understanding of how and why the intervention works was not explored nor were their subsequent personal plans for improvement. These concerns with focusing only on task-related effects (e.g. prescribing errors) have recently been well described by Ajjawi et al. [42], albeit in higher education, and suggest that subsequent studies consider qualitatively exploring learners’ sense making when engaging these types of interventions.

This intervention was labour intensive, using an analogue system for data collection and providing the feedback to 20 interns in five 10-week terms. We are unable to evaluate the cost effectiveness of our intervention. Electronic prescribing may increase the ease of data collection and efficiency of any intervention. However, hospitals utilise extensive resources undertaking traditional quality assurance prescribing audits with generalised, generic feedback which remains largely ineffective in changing prescribing behaviour without putting it into context for the individual prescriber. It is unclear if digital systems will deliver significant benefits in terms of enabling effective prescriber feedback.

### Future work

To understand how best self and peer feedback can be translated into practice, further in-depth qualitative controlled evaluation is required of how and why the intervention impacted on certain individual prescribers’ safety of prescribing, regardless of how much practical experience they had had during their intern year. This intervention may be equally beneficial for more experienced medical staff and other non-medical prescribers. Finally, there will be value in drawing on the implementation science principles of spread and scaling-up [43], to generate broader sustainable strategies to improve junior doctor prescribing through feedback interventions to reduce errors in hospitals.

## Conclusion

Our constructivist feedback theory (Feedback Mark 2) pre-post intervention study resulted in a reduction in prescribing errors, which is a global, national healthcare objective and individual goal of the interns who participated. This approach offered a systematic and innovative way to design a prescribing feedback intervention. This included opportunities for an orientation to standards, self-assessment and the utilisation of individuals prescribing examples, which may not have previously been considered. Traditional hospital-based quality assurance audits with general feedback will remain ineffective in changing behaviour without personalising feedback to the individual. Learner centred and dynamic descriptive feedback with an agreed plan could significantly improve prescribing safety. Further research to examine generalisability and sustainability of this feedback intervention design is required.

## Electronic supplementary material

Below is the link to the electronic supplementary material.

Supplementary Material 1

Supplementary Material 2

## Data Availability

The datasets generated and/or analysed during the current study are not publicly available due the risk of compromising individual privacy but are available from the corresponding author on reasonable request.
