# Supplementary material for: Can a novel constructivist theory-informed feedback intervention reduce prescribing errors ? A pre-post study
Source: BMC Med Educ. 2023 Mar 7;23:150. doi: 10.1186/s12909-023-04095-6 (PMC9993618; doi:10.1186/s12909-023-04095-6)
Supplement: Supplementary file 1 — Supplementary Material 1 [file 12909_2023_4095_MOESM1_ESM.docx]

# **Appendix One** –

# Resident Prescribing Competency Evaluation and Feedback, Safety and Quality Development Tool

| **Competency** | **Tick** | **Rarely** | **Sometimes** | **Usually** | **Consistently** | **Comments / examples/ recommendations** |
| --- | --- | --- | --- | --- | --- | --- |
| **Medication history**  (drug, dose, complete) | S |  |  |  |  |  |
|  | P |  |  |  |  |  |
| **Patient ID**  (all charts & printed name) | S |  |  |  |  |  |
|  | P |  |  |  |  |  |
| **ADR/ Allergy** (documented drug + rxn or un/ nil known) | S |  |  |  |  |  |
|  | P |  |  |  |  |  |
| **VTE prophylaxis**  (appropriate) | S |  |  |  |  |  |
|  | P |  |  |  |  |  |
| **Drug Name**  (clear & generic) | S |  |  |  |  |  |
|  | P |  |  |  |  |  |
| **Indication**  (present) | S |  |  |  |  |  |
|  | P |  |  |  |  |  |
| **AMS**  (used/ acted on) | S |  |  |  |  |  |
|  | P |  |  |  |  |  |
| **Route**  (clear & correct) | S |  |  |  |  |  |
|  | P |  |  |  |  |  |
| **Drug dose**  (clear & correct) | S |  |  |  |  |  |
|  | P |  |  |  |  |  |
| **Frequency & admin times**  (correct + entered) | S |  |  |  |  |  |
|  | P |  |  |  |  |  |
| **PRN dosing**  (frequency + Max dose) | S |  |  |  |  |  |
|  | P |  |  |  |  |  |
| **Ceasing medications**  (order,admin,sign,date) | S |  |  |  |  |  |
|  | P |  |  |  |  |  |
| **Signature** | S |  |  |  |  |  |
|  | P |  |  |  |  |  |

| **Competency** | **Standard of care and rationale for that standard** |
| --- | --- |
| **1. Medication History**  (drug, dose, complete) | ***Medicines contribute to up to 20% admissions and continuity of care of chronic conditions.***  An initial list of medicines taken prior to admission should be in the medical chart or on medication action plan. It should include names, doses, and frequencies. Duration of therapy is useful if related to a presenting complaint. Includes any recent changes that may link to admission. |
| **2. Patient ID**  – all charts & printed name | ***Patients have been administered all of another’s medicines because wrong ID was attached.***  All pages of the NIMC or ancillary charts have the correct computer generated ID label OR patients name, UR number, address and DOB. Patients name is printed underneath the IB label – this is considered as a cognitive prompt to prescriber re name the chart is intended for and is a cross check in case an ID label has been applied to a chart intended for a different patient. |
| **3. ADR/ Allergy**  documented including reaction | ***Patients re-­‐exposed to the same or similar class drug to which they had a previous ADR can cause severe adverse events.***  Before prescribing the prescriber should check and or identify any previously known or recorded ADRs or allergies. If none are known tick that box. If unable to verify any previous ADR (unconscious patient etc.) tick “unknown”. The NIMC should include the drug and reaction and idea of date that it occurred. |
| **4. VTE prophylaxis**  (appropriate) | ***Over 5000 patients die from preventable hospital associated VTE each year. Adverse bleeding events occur when these risks aren’t appropriately managed and considered.***  Consideration of a patient’s VTE risk and or any contraindication should be documented on the chart and appropriate therapy prescribed according with QH, RBWH or local guidelines. |
| **5. Drug Name**  (present, clear & generic) | ***Unclear drug names result in patients receiving unintended medication and missing essential medicine.*** Ideally always use generic (exception, SR opiates, Mylanta, insulins, combination inhalers |
| **6. Indication** present | ***Medications may be inadvertently stopped, changed or withheld if other members of the medicine team are unaware of intended indication:*** Communication between prescribers and nurses and pharmacists ensures appropriate medication management, continuity, and patient education. |
| **7. AMS**  (used/ acted on) | ***Inappropriate ant-­‐ microbial prescribing leads to resistance and adverse events such as c.diff:***  Prescribers should become familiar with green / yellow and red traffic light antimicrobials.  Prescribers should know how to use AMS and seek guidance (electronically or via pharmacy or AMS/ ID teams to ensure optimal use of antimicrobials. Recommendations should be acted on |
| **8. Route**  (present, clear & correct) | ***The absence of any instruction or misinterpretation of unclear routes ie sl/ sc have lead to drugs being given by the incorrect route.*** Sub cut is safer than sc) and Sub Ling (safer than sl) |
| **9. Drug dose**  (present, clear & correct) | ***The most frequent medication errors are associated with drug dose. These are either where it is inappropriate for the patient (age, size, renal function, concomitant therapy, physiological parameters) missing or unclear.*** Clear and correct dose for that patient with that condition is required***. In paediatrics/ neonates ensuring weight based dosing is essential.*** |
| **10. Frequency & admin times** (present,  clear & correct) | ***Dosing frequency may be wrong for a patient’s parameters ie reduced renal function, “pseudo-­‐latin hieroglyphics” (ie*** q6h, 4 hrly, od, d ***)can commonly misinterpreted by nursing staff – who may enter the wrong administration times or other medical staff.*** Consider dose adjustment for your individual patient and write out daily/ evening or accepted abbreviations bd, tds, qds |
| **11. PRN dosing**  – frequency + Max dose | ***PRN / as required dosing is intended to ensure that nursing staff tailor symptom relieving medication to a patients specific requirements whilst minimising adverse events. TDS may be interpreted as every 8 hours or three doses within 1 hour each day.*** For a drug that can be given in multiple daily doses use hourly (ie: 4 hourly) and include a maximum quantity every 24 hours (taking into account any regular drugs ie: oxycodone or paracetamol) |
| **12. Ceasing medications**  (order,admin,sign,date) | ***Not clearly ceasing a medication may lead to inadvertent continuation during an admission or being unnecessarily continued on discharge.*** Prescribers should cross through BOTH the prescribing and administration section, and initial and date when that is done. Ideally best practice includes explaining why the medication is changed ie “Ceased IDC, 27/3, reduced renal fct” |
