# Supplementary material for: Can a novel constructivist theory-informed feedback intervention reduce prescribing errors ? A pre-post study
Source: BMC Med Educ. 2023 Mar 7;23:150. doi: 10.1186/s12909-023-04095-6 (PMC9993618; doi:10.1186/s12909-023-04095-6)
Supplement: Supplementary file 2 — Supplementary Material 2 [file 12909_2023_4095_MOESM2_ESM.docx]

Appendix Two **Adapted National Inpatient Medication Chart Prescribing Audit Tool**

| **Patient Identification** |
| --- |
| Total number of medication charts (i.e. charts in use) |
| n current medication chart pages with **complete** ID (handwritten / label) |
| n current medication chart pages **with** ID label |
| **Adverse Drug Reaction (ADR) Details, Alerts & Errors** |
| n ADR documented - nil known / unknown ticked |
| n previous ADRs |
| n ADR where generic medication names documented |
| n ADR reaction details documented |
| Similar class of medication prescribed (Y/N) |
| n pages with ADR alert stickers (if previous ADR) |
| **Warfarin Dosing & Administration** |
| Warfarin order (Y/N) |
| n of warfarin orders with indication documented |
| n of warfarin orders with target INR range documented |
| **Venous Thromboembolism (VTE) Prophylaxis** |
| VTE Risk Assessment documented on any medication chart (Y/N) |
| VTE Prophylaxis prescribed (Y/N) |
| VTE Prophylaxis prescribed in VTE section (Y/N/NA) |
| **Duplicated orders** |
| Duplicated orders (Y/N) |
| **Prescribing** |
| n 'once only', stat, telephone orders |
| n current regular medications + variable, warfarin + ceased orders |
| n current PRN medications + ceased PRN orders |
| **Prescribing: 'Once Only', Stat, Telephone, Regular & PRN** |
| n medications with unacceptable trade name only (all orders) |
| n medications with name unclear including error prone abbreviations (all orders) |
| n medications with route unclear including error prone abbreviations (all orders) |
| n medications with route incorrect or missing (all orders) |
| n medications with dose unclear including error prone abbreviations (all orders) |
| n medications with dose incorrect or missing (all orders) |
| **Prescribing: Regular** |
| n frequency unclear including error prone abbreviations (regular only) |
| n frequency incorrect or missing (regular ) |
| n administration times entered by prescriber (regular orders) |
| n administration times not correlating with frequency entered by nurse (regular orders) |
| n administration times not correlating with frequency entered by prescriber (regular orders) |
| n sustained release medications (regular orders only) |
| n sustained release medication with SR box ticked |
| n intermittent medications prescribed (i.e. weekly, fortnightly, twice weekly) |
| n intermittent medication not boxed by prescriber |
| n regular orders with an indication documented |
| n ceased orders (regular & PRN) |
| n orders ceased according to hospital policy (cross order + admin + sign + indication) |
| **Prescribing Errors Specific to PRN Orders** |
| n missing PRN frequencies |
| n unclear PRN frequencies including error prone abbreviations |
| n PRN orders with an indication documented |
| n PRN orders with a maximum dose documented |
| **Frequency of Prescriber Signature and Printed Name** |
| n of orders signed by prescriber (all orders) |
| n of orders where prescriber name is clear (all orders) |
